# HIV/Human herpesvirus co-infections: Impact on tryptophan-kynurenine pathway and immune reconstitution

**DOI:** 10.1371/journal.pone.0186000

**Published:** 2017-10-09

**Authors:** Siew Hwei Yap, Noor Kamila Abdullah, Megan McStea, Kozo Takayama, Meng Li Chong, Elisa Crisci, Marie Larsson, Iskandar Azwa, Adeeba Kamarulzaman, Kok Hoong Leong, Yin Ling Woo, Reena Rajasuriar

**Affiliations:** 1 Centre of Excellence for Research in AIDS (CERiA), University of Malaya, Kuala Lumpur, Malaysia; 2 Department of Pharmaceutics, Hoshi University, Tokyo, Japan; 3 Department of Clinical and Experimental Medicine, Linköping University, Linköping, Sweden; 4 Department of Medicine, University of Malaya, Kuala Lumpur, Malaysia; 5 Department of Pharmacy, University of Malaya, Kuala Lumpur, Malaysia; 6 Department of Obstetrics & Gynecology, University of Malaya, Kuala Lumpur, Malaysia; 7 Peter Doherty Institute for Infection and Immunity, Melbourne University, Victoria, Australia; Hospital Juan Fernández, ARGENTINA

## Abstract

**Background:**

Co-infections with human herpesvirus (HHV) have been associated with residual chronic inflammation in antiretroviral (ART)-treated human immunodeficiency virus (HIV)-infected individuals. However, the role of HHV in modulating the tryptophan-kynurenine pathway and clinical outcomes in HIV-infected individuals is poorly understood. Thus, we investigated the seroprevalence of four common HHVs among treated HIV-infected participants and their impact on kynurenine/tryptophan (K/T) ratio and long-term CD4 T-cell recovery in HIV/HHV co-infected participants.

**Method:**

In this cross-sectional study, HIV-infected participants receiving suppressive ART for a minimum of 12 months were recruited from the University Malaya Medical Centre (UMMC), Malaysia. Stored plasma was analyzed for CMV, VZV, HSV-1 and HSV-2 IgG antibody levels, immune activation markers (interleukin-6, interferon-γ, neopterin and sCD14), kynurenine and tryptophan concentrations. The influence of the number of HHV co-infection and K/T ratio on CD4 T-cell recovery was assessed using multivariate Poisson regression.

**Results:**

A total of 232 HIV-infected participants were recruited and all participants were seropositive for at least one HHV; 96.1% with CMV, 86.6% with VZV, 70.7% with HSV-1 and 53.9% with HSV-2. K/T ratio had a significant positive correlation with CMV (rho = 0.205, *p* = 0.002), VZV (rho = 0.173, *p* = 0.009) and a tendency with HSV-2 (rho = 0.120, *p* = 0.070), with CMV antibody titer demonstrating the strongest modulating effect on K/T ratio among the four HHVs assessed in SOM analysis. In multivariate analysis, higher K/T ratio (*p* = 0.03) and increasing number of HHV co-infections (*p*<0.001) were independently associated with poorer CD4 T-cell recovery following 12 months of ART initiation.

**Conclusion:**

Multiple HHV co-infections are common among ART-treated HIV-infected participants in the developing country setting and associated with persistent immune activation and poorer CD4 T-cell recovery.

## Introduction

Infection with human immunodeficiency virus (HIV) is still associated with a high degree of morbidity and mortality in developing countries partly due to the limited access to antiretroviral therapy (ART) [1]. While the survival rates among those on treatment have improved, a subset of patients (ranging from 15% to 40%) continue to experience sub-optimal CD4 T-cell recovery [2], which in turn has been associated with an increased risk of serious non-AIDS events (SNAEs) [2,3]. Thus, understanding the mechanisms modulating immune recovery in resource limited setting remains a high priority in the management of HIV.

Chronic immune activation and inflammation are associated with SNAEs and sub-optimal CD4 T-cell recovery [2,4,5]. Latent co-infections have been suggested to have a more significant influence in driving chronic immune activation in developing compared to developed country setting where endemic infections are generally more prevalent and ART is initiated late [6–8].

Human herpes viruses (HHVs) are among the most prevalent sexually transmitted infections (STIs) particularly among immunocompromised individuals in the developing world [9–11]. In rural South Africa, the prevalence of cytomegalovirus (CMV), varicella zoster virus (VZV), human simplex virus-1 (HSV-1) and human simplex virus 2 (HSV-2) in HIV-infected subjects were found to be 100%, 87%, 98% and 87%, respectively [9]. Besides, co-infections with CMV, HSV, human herpesvirus 8 (HHV8), and VZV, have all been associated with residual chronic inflammation in HIV-infected participants receiving suppressive ART [7,12–15]. CMV particularly has been shown to be directly associated with serious non-AIDS events [8], cardiovascular morbidity [16], stroke [17], poorer neurocognitive performance [18] and frailty [19] in HIV while HSV and VZV infections have been associated with an increased risk of stroke in the HIV [20] and general population [21]. An increasing burden of multiple HHV co-infections were also found to be associated with increased atherosclerosis in the Multicenter AIDS Cohort Study (MACS) [20]. Thus, targeting the immune activation pathways associated with HHV co-infections in HIV may be a potential therapeutic option in reducing morbidity in HIV-infected participants in resource limited settings.

Interferon-γ is one of the main antiviral cytokines produced following HHV infections [22,23]. IFN-γ induces the indoleamine-2,3-deoxygenase (IDO) enzyme, the rate limiting enzyme in the kynurenine pathway of tryptophan catabolism [24]. IDO activity which is measured by changes in the ratio of tryptophan and its metabolite, kynurenine (herein referred to as K/T ratio), remains elevated in chronic HIV despite ART [25–27] though early ART initiation in primary HIV has been shown to normalize levels [27]. Persistently elevated IDO activity is associated with increased mortality, reduced T-cell proliferation, poorer CD4 T-cell reconstitution of the gut-associated lymphoid tissue (GALT), and neurological dysfunction [27–31]. While the role of HHV and particularly CMV in driving T-cell activation and senescence is well described in HIV [32–34], its specific role in modulating the IFN-driven IDO pathway in the setting of HIV is less well studied.

Given the high rate of HHV infections in the developing country setting and its association with SNAEs, we hypothesized that HHV co-infection in HIV-infected patients may be an important modulator of K/T ratio and clinical outcome. Therefore, the aims of this study were to 1) measure the seroprevalence of four common HHVs specifically CMV, VZV, HSV-1 and 2 among treated HIV-infected participants; 2) explore the association of IDO activity (measured as K/T ratio) with HHV seropositivity and other markers of immune activation; and 3) assess the influence of HHV seroprevalence and K/T ratio on long-term CD4 T-cell recovery in HIV-infected participants receiving ART.

## Methods

### Study population

Participants were recruited among HIV-infected patients attending the Infectious Diseases Unit at University Malaya Medical Centre (UMMC), Malaysia for their routine follow-up. Participants fulfilling the following inclusion criteria were approached to participate in the study; men or women aged at least 18 years, receiving ART and having achieved controlled viremia (defined as HIV RNA <50 copies/ml) for at least 12 months following ART commencement. Clinical data on HIV transmission-risk behavior, duration of ART, current ART regimen and all CD4 T-cell count and HIV RNA measures from treatment initiation to the date of recruitment were abstracted from patient medical records. All participants provided written informed consent. The study protocol was approved by the University Malaya Medical Centre Ethics Review Board (MEC Ref. No 896.32).

### Human herpes virus serology

Whole blood was collected following overnight fasting and plasma separated as previously described [35]. Plasma samples were stored at -80°C until further analysis. HSV-1 and 2 IgG levels were analyzed using HerpeSelect® enzyme-linked immunosorbent assay (ELISA) IgG kits (Focus Diagnostics, Cypress, CA, USA) as previously described [36]. The Elecsys® (Roche, Switzerland) and Siemens Enzygnost® (Siemens Healthcare GmbH, Germany) kits were used to quantify CMV and VZV IgG class antibodies, respectively. Sample optical density readings were compared with reference cutoffs to determine positive, negative, or equivocal status. A cut-off of >1.1 was considered as HSV-1 and HSV-2 seropositive. A cut-off of >1.0 and a cut-off of >0.2 were used for CMV seropositivity and for VZV seropositivity, respectively, according to the manufacturer’s instructions. Equivocal samples were considered as negative.

### Measurement of IDO activity

Tryptophan and kynurenine levels were measured by using liquid chromatography-tandem mass spectrometry (LC-MS/MS) as described in Huang *et al*. [37]. Briefly, trifluoroacetic acid was added into the plasma samples for protein plasma precipitation before analysis. A total of 5μl sample was injected into the LC-MS/MS system (Agilent, 6400 series). The HPLC conditions were as follows: the column was a Synergi Polar RP column (75 x 4.6mm) from Phenomenex (CA, USA) and the mobile phase was composed of formic acid in ultrapure water and a mixture of methanol and acetonitrile (7% v/v) with gradient program. The flow rate was set at 1.0 ml/min and the run time for each sample was 6 min. The LC-MS/MS method was validated according to the International Council for Harmonization (ICH) guidelines. K/T ratio was calculated by dividing the concentration kynurenine to that of tryptophan, both measured in ng/ml.

### Measurement of immune activation markers

Plasma neopterin and sCD14 were measured using conventional sandwich ELISA platform (Thermo Scientific, Hennigsdorf and R & D Systems, USA, respectively) according to the manufacturer’s instructions. The markers IFN-γ and IL-6 were measured using the enhanced sensitivity cytometry bead array (CBA) (Becton Dickinson, San Jose, CA) according to the manufacturer’s instructions and acquired on a FACS Canto II (Becton Dickinson, San Jose, CA). Analysis was performed using the FCAP Array software (Becton Dickinson, San Jose, CA).

### Statistical analysis

Statistical analysis was performed using the Statistical Package for the Social Sciences (SPSS) 20.0 for Windows (IBM, United States) and Stata Statistical Software: Release 14 (StataCorp LP, College Station, TX). Correlations between immune activation markers and K/T ratio were tested by non-parametric Spearman rank correlation coefficients. Comparison of immune activation markers in individuals with different numbers of HHV co-infection were assessed using Poisson Regression models adjusting for age, gender and ethnicity as the background for each participant is different. Results were considered significant at *p*-value <0.05. Population averaged panel data models based on generalized linear regression were used on both a univariate and multivariate basis to assess the risk factors associated with CD4 T-cell recovery between the 0 to 12 month period and >12 months following ART initiation. A cut-off of 12 months was chosen as previous studies have demonstrated that CD4 T-cell increase following ART initiation exhibits a profile of exponential increase in the first 6–12 months followed by a linear increase thereafter [38–40]. The initial exponential CD4 T-cell increase is driven by the redistribution of T-cells from the lymphoid tissues following suppression of immune activation with ART while the second linear phase of increase is associated with de novo production of T-cells [38]. Log transformations of covariates included in the model were applied where appropriate. CD4 T-cell counts for the assessment of CD4 T-cell recovery only included periods of confirmed viral suppression and observations were censored when; (1) treatment interruption of > 2 weeks occurred; (2) frequency of plasma HIV RNA level determinations declined to <1/year; or (3) participants experienced virological rebound defined as two consecutive plasma HIV RNA >1000 copies/ml. Variables with p-value of <0.250 or those covariates considered clinically important (age, gender and ethnicity) were included in the multivariate models with forward and backward stepwise regression techniques as well as variance inflation factor (VIF) testing used to develop the final model presented in this paper. Interactions were tested and considered significant at the 0.05 level.

### Self-organizing map

Viscovery® SOMine version 5.0 (Eudaptics Software GmbH, Vienna, Austria) was used for the Kohonen’s self-organizing map (SOM) analysis [41] clustering the positive or negative of four HHVs and featuring K/T ratio as an outcome. Briefly, SOM is a feedforward-type neural network learning model comprising one input layer and one output layer, and the array of nodes is located in the output layer. The SOM algorithm is based on unsupervised, competitive learning. The network ultimately associates the output nodes with groups or patterns of input vectors by repeating the learning. The SOM was used to generate feature maps and clustering of four HHVs and associating K/T ratio. For mapping, the weight (w) of four markers was assigned a value of 1 (w = 1), but no weight (w = 0) was adapted for K/T ratio. For graphical display, the four-dimensional characteristic spaces (representing four HHVs) and one outcome (K/T ratio) were projected onto an output layer comprising 500 nodes. The level of K/T ratio can be studied from the density of color shades in the nodes network, where areas of red and yellow indicate a higher value of K/T ratio and blue and green indicate lower values of K/T ratio.

## Results

### Participant characteristics

A total of 232 treated HIV-infected participants were included in the analyses. The majority were male (85.3%) and of Chinese ethnicity (75.9%). At the time of enrolment, the median age was 37 years (interquartile range [IQR], 32–43) and the median baseline CD4 T-cell count was 110 cells/μL (IQR, 27–232 cells/μL). More than two-thirds (68.1%) of the cohort had a history of AIDS-defining illness while the median (IQR) current CD4 T-cell counts were 583 cells/uL (IQR, 427–763 cells/uL). A high prevalence of seropositivity for HHV (CMV = 96.1%, VZV = 86.6%, HSV-1 = 70.7%, HSV-2 = 53.9%,) was observed in this cohort (Table 1) with 97% reporting seropositivity for multiple pathogens. The number of HHV co-infections were quantified to reflect the cumulative number of HHV seropositivity in a participant with 3% having one HHV co-infection, 17.2% with 2, 49.1% with 3 and 30.6% seropositive for all 4 HHVs. All participants were seropositive for at least one HHV.

**Table 1:** Characteristics of human immunodeficiency virus (HIV)-infected Malaysians included in this study.

Characteristics	Values
Gender, n (%)	
Male	198 (85.3)
Female	34 (14.7)
Ethnicity, n (%)	
Chinese	176 (75.9)
Indian	21 (9.1)
Malay	35 (15.1)
HIV Transmission Risk, n (%)	
Homosexual contact	48(20.7)
Injection drug use	6 (2.6)
Heterosexual contact	130 (56.0)
Heterosexual contact & IDU	3 (1.3)
Receipt of blood/blood products	1 (0.4)
Not specified	44 (19.0)
Smoking Status, n (%)	
No	86 (37.1)
Current smoker	98 (42.2)
Previous Smoker	1 (0.4)
Not recorded	47 (20.3)
History of Syphilis, n (%)	
Negative	148 (63.8)
Positive	58 (25.0)
Not recorded	26 (11.2)
ART Treatment Regimen, n (%)	
NNRTI-based	225 (97.0)
PI-based	7 (3.0)
History of AIDS-defining illness*, n (%)	
No	74 (31.9)
Yes	158 (68.1)
Age, year	37 (32–43)
hsCRP, mg/L	0.26 (0.10–0.50)
Baseline CD4 T-cell count, cells/uL	110 (26.5–323)
Baseline Viral Load, copies/mL	102347 (63208–269175)
Current CD4 T-cell count, cells/uL	583 (428–763)
Current CD4:CD8 ratio	0.64 (0.43–0.89)
Duration of ART, year	7.0 (4.6–10.3)
Neopterin, nmol/mL	13.18 (10.41–16.58)
sCD14, x10^6^ pg/mL	1.98(1.63–2.30)
K/T ratio	0.0219 (0.0168–0.0260)
Kynurenine concentration (ng/ml)	273.46 (222.81–336.38)
Tryptophan concentration (ng/ml)	12777.33 (10742.53–14785.59)
Hepatitis B Antigen^#^	
Negative	209 (90.1)
Positive	14 (6.0)
Hepatitis C Antibody^#^	
Negative	210 (90.5)
Positive	18 (7.8)
HSV-1 seropositive, n (%)	164 (70.7%)
Median IgG levels (IQR), AU	4.200 (0.335–4.894)
HSV-2 seropositive, n (%)	125 (53.9)
Median IgG levels (IQR), AU	1.666 (0.340–6.097)
CMV seropositive, n (%)	223 (96.1)
Median IgG levels (IQR), AU	500.000 (247.400–500.000)
VZV seropositive, n (%)	201 (86.6)
Median IgG levels (IQR), AU	1.200 (0.720–1.590)
Number of HHV co-infections, n (%)	
1	7 (3.0)
2	50 (17.2)
3	114 (49.1)
4	71 (30.6)

Data are number (%) of participants or median (interquartile range).

^#^ There are 9 participants with missing data for Hepatitis B and 4 missing for Hepatitis C.

* AIDS-defining illnesses included pneumocystis jiroveci pneumonia, esophageal candidiasis, cervical cancer, cryptococcal meningitis, cryptosporidiosis, cytomegalovirus disease, histoplasmosis, Kaposi sarcoma, mycobacterium tuberculosis, mycobacterium avium complex progressive multifocal leukoencephalopathy, salmonella septicemia, and toxoplasmosis of brain^.^

Abbreviation: IDU, injection drug user; ART, antiretroviral therapy; NNRTI, non-nucleoside reverse transcriptase inhibitors; PI, protease inhibitor; AIDS, acquired immune deficiency syndrome; hsCRP, C-reactive protein; K/T ratio, ratio of kynurenine to tryptophan; HSV-1, human simplex virus-1; HSV-2, human simplex virus-2; CMV, cytomegalovirus; VZV, varicella zoster virus.

### K/T ratio correlates with HHV co-infections and immune activation markers

Correlations between K/T ratio and HHV antibody levels, as well as markers of immune activation were explored (Table 2) with differences between seropositive and negative participants shown in S1 Table. We found that K/T ratio was positively correlated with CMV antibody levels (rho = 0.205, *p* = 0.002) and VZV antibody levels (rho = 0.173, *p* = 0.009) with a tendency of correlation with HSV-2 antibody levels (rho = 0.120, *p* = 0.070). Among the other markers of immune activation, K/T ratio significantly correlated with IL-6, neopterin, sCD14 and hsCRP.

**Table 2:** Correlation of K/T ratio with IL-6, IFN-γ, neopterin, hsCRP, sCD14, HSV-1 antibody level, HSV-2 antibody level, CMV antibody level and VZV antibody level.

Variable	K/T ratio	
	Rho	*p*-value
HSV-1 antibody level	-0.099	0.134
HSV-2 antibody level	0.120	0.070
CMV antibody level	0.205	0.002*
VZV antibody level	0.173	0.009*
IL-6	0.338	0.000*
IFN-γ	0.142	0.111
Neopterin	0.628	0.000*
hsCRP	0.169	0.011*
sCD14	0.330	0.000*

*Significance at p<0.05^.^

Abbreviation: hsCRP, C-reactive protein; K/T ratio, ratio of kynurenine to tryptophan; HSV-1, human simplex virus-1; HSV-2, human simplex virus-2; CMV, cytomegalovirus; VZV, varicella zoster virus; sCD14, soluble CD14; IL-6, interleukin-6; IFN-γ, interferon-γ.

**Fig 1:**
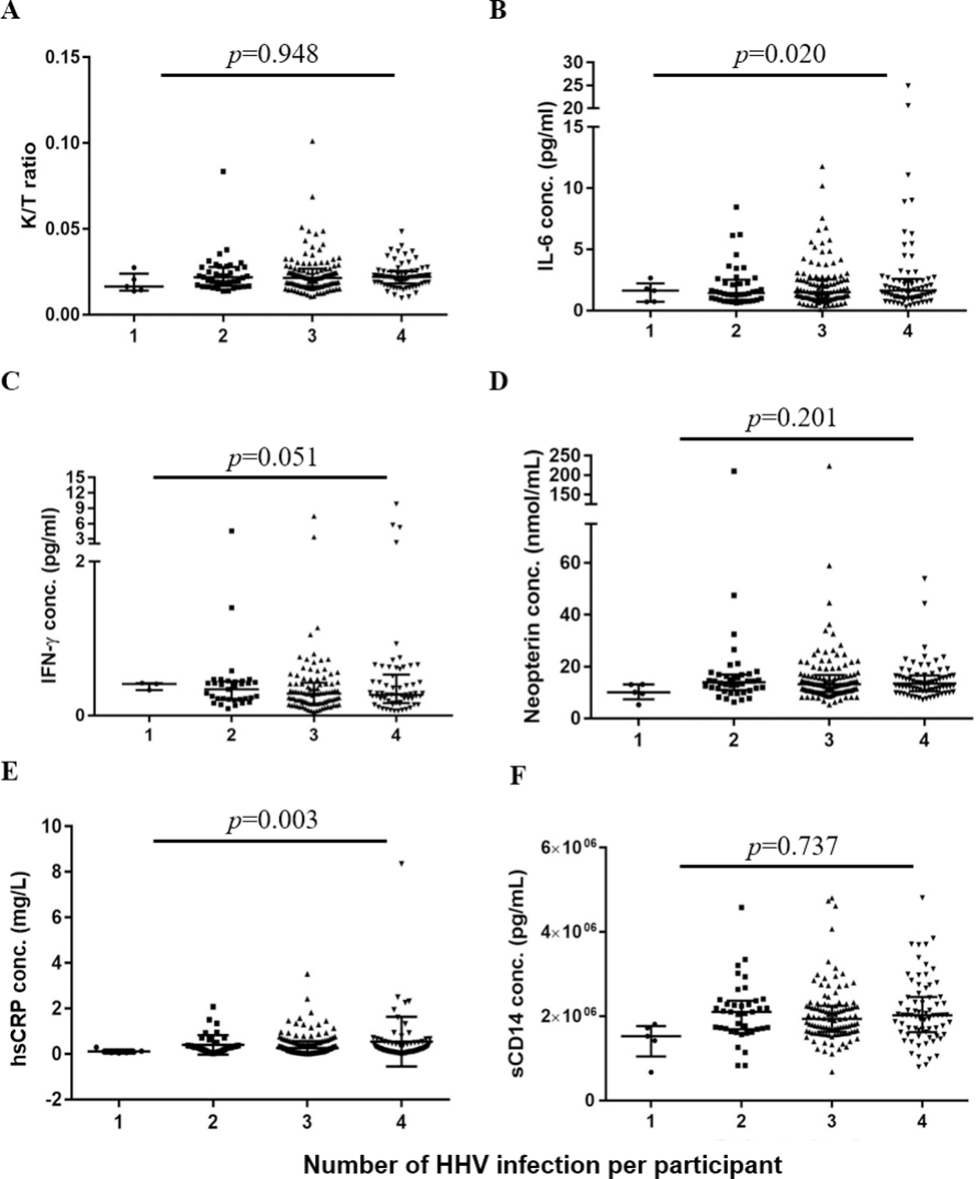
**Comparisons in K/T ratio (A), IL-6 (B), IFN-γ (C), neopterin (D), hsCRP (E) and sCD14 (F) in groups with increasing number of HHV co-infections.** The p-values indicate statistical significance following Poisson regression modelling, adjusted for age, gender and ethnicity. The error bars indicate the median(IQR) for each group. The numbers in the x-axis indicate the total number of HHV co-infections per participant. *Significance at p<0.05.

We investigated whether an increasing number of HHV co-infection in a participant had an impact on K/T ratio and the other immune activation markers (Fig 1). Using Poisson regression analysis and adjusting for age, gender and ethnicity, a significantly positive association was found between the number of HHV co-infections and hsCRP and IL-6, with a tendency towards significance observed with IFN-γ. This indicates that increasing number of HHV co-infections are associated with higher levels of systemic inflammation in virologically suppressed HIV-infected participants. Increasing number of HHV co-infections was however not associated with increasing K/T ratio.

### CMV seropositivity had the strongest modulating effect on K/T ratio

**Fig 2:**
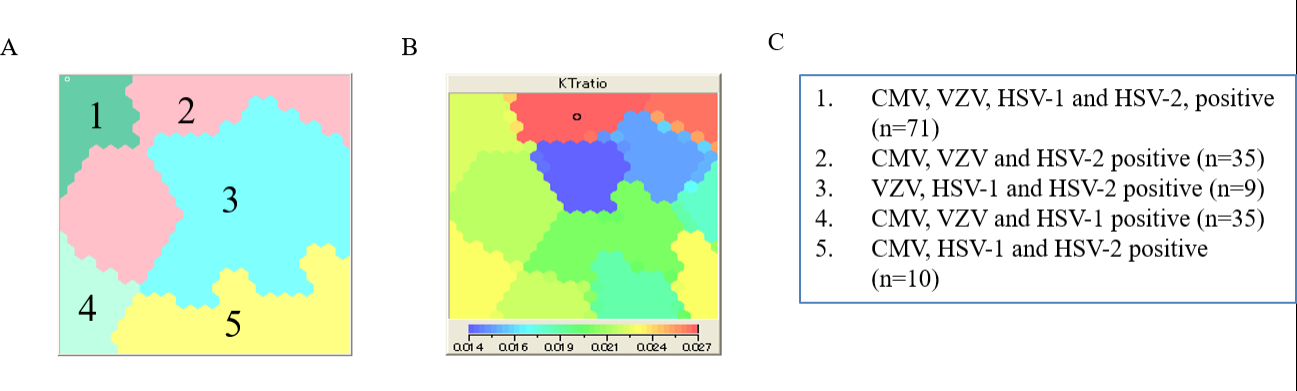
Clustering of human herpes virus (cytomegalovirus, CMV; varicella zoster virus, VZV; Herpes Simplex Virus-1, HSV-1; Herpes Simplex Virus-2, HSV-2) by self-organizing map (SOM) on Kynurenine/ Tryptophan ratio (K/T ratio). (A) Clustering map of CMV, VZV, HSV-1 and HSV-2; (B) Feature map of K/T ratio, which was associated with no weight in the SOM analysis; (C) Combination of HHVs for the cluster A.

We next explored which combinations of HHV infections had the greatest effect on modulating K/T ratio in a participant (Fig 2) utilizing the SOM approach. Fig 2A shows the different combinations of HHVs measured in the participants, and this corresponds to the areas describing the different intensities of K/T ratio in Fig 2B. We analyzed all possible combinations for the four HHVs tested and found that participants seropositive for CMV, VZV and HSV-2 were clustered in areas indicating the highest intensity of K/T ratio (Fig 2B, red area). Additionally, as shown in Fig 2B, the areas indicating the lowest value of K/T ratio (blue and green areas) corresponded with clusters in Fig 2A where none of the participants were seropositive for CMV. Notably, there were also different intensities of K/T ratio within individual clusters of HHV combinations (e.g. cluster 2, 3 and 5) suggesting the role of other unmeasured factors contributing to K/T ratio modulation in these study participants. These data imply that there is a complex modulation of the kynurenine-tryptophan pathway in the presence of multiple HHV infections and among the four HHVs tested, co-infections involving CMV had the strongest modulating effect on K/T ratio.

### HHV co-infection and K/T ratio influences CD4 T-cell recovery

To assess the factors associated with early and late immune recovery, we performed generalised linear estimation on our panel data which recorded regular assessments of CD4 T-cell counts and HIV RNA which extended beyond 60 months in most cases (Table 3). Median follow-up duration for ART-mediated viral suppression was 189 (IQR,140–266) months. Participants contributed a median of three CD4 T-cell count observations/patient (IQR, 3–4) in the model for 0–12 months post ART and a median of 11 CD4 T-cell count observations/patient (IQR, 7–15) in the model assessing risk factors >12 months post ART. An effect modifying the interaction between age and the K/T ratio (log transformed) was found and incorporated into the model. After adjustment for age, gender, ethnicity and all other univariately significant variables and interactions, we found that a higher K/T ratio (log transformed) was associated with an increase in CD4 T-cell counts. This translated to an average increase of 5 cells/μL increase for every 10% increase in K/T ratio in the early phase of recovery from 0–12 months post-ART. Age and K/T ratio were found to have a statistically significant interaction effect. Other factors significantly associated with early CD4 T-cell recovery included a history of AIDS-defining illness, younger age, shorter duration on ART, higher log baseline viral load (time-dependent), higher baseline CD4 T-cell and higher CD8 T-cell counts (time-dependent).

**Table 3:** Risk factors associated with CD4 T-cell recovery between the 0 to 12 month period following ART initiation.

Variable	Univariate β-coefficient	*p*-value	Multivariate β-coefficient	*p*-value
Age	-1.98 (-3.14, -0.81)	0.001*	-11.91 (-18.10, -5.72)	<0.001*
Gender				
Male				
Female	19.31 (-14.84, 53.47)	0.268	-0.16 (-19.81, 19.50)	0.988
Ethnicity		0.327		0.504
Chinese				
Indian	14.20 (-27.79, 56.19)	0.507	10.93 (-10.70, 32.55)	
Malay	23.77 (-9.04, 56.59)	0.327	6.89 (-10.41, 24.19)	
History of AIDS-defining illness		0.017*		0.011*
No				
Yes	31.79 (5.75, 57.84)		17.24 (3.93, 30.55)	
Hep C antibody	-37.71 (-91.11, 15.68)	0.166		
Hep B antigen	-19.63 (-65.94, 26.67)	0.406		
Number of HHV co-infections		0.048*		
1				
2	-35.42 (-112.50, 41.66)	0.368		
3	13.05 (-60.40, 86.51)	0.728		
4	4.38 (-70.91, 77.86)	0.927		
hsCRP (Log)	7.56 (-2.52, 17.64)	0.142		
Duration of ART	-4.50 (-7.99, -1.01)	0.011*	-2.92 (-5.07, -0.77)	0.008*
Baseline Viral Load (Log)	-28.27 (-37.04, -19.49)	<0.001*	13.21 (7.0, 18.73)	<0.001*
Viral Load (Log)	-15.64 (-18.70, -12.58)	<0.001*	-13.61 (-15.25, -11.96)	<0.001*
Baseline CD4 T-cell count	1.00 (0.94, 1.06)	<0.001*	1.00 (0.94, 1.06)	<0.001*
CD8 T-cell count (Time-dependent)	0.117 (0.91, 0.14)	<0.001*	0.04 (0.03, 0.06)	<0.001*
K/T ratio (Log)	-42.04 (-75.06, -8.47)	0.014*	129.95 (60.05, 199.85)	<0.001*
**INTERACTION**				
K/T ratio (Log) X Age			-3.13 (-4.81, -1.46)	<0.001*

*Significance at p<0.05.

Abbreviation: hsCRP, C-reactive protein; ART, antiretroviral therapy; K/T ratio, ratio of kynurenine to tryptophan.

In analyzing factors associated with CD4 T-cell recovery beyond 12 months on ART (Table 4), an increase in K/T ratio (log transformed) was associated with a decrease in CD4 T-cell counts. Thus, for every 10% increase in K/T ratio, there was a 1 cell/μL decline in CD4 T-cell count >12 months post-ART. Additionally, an increasing number of HHV co-infections compared to participants with a single HHV co-infection was found to be associated with on average 137–160 cells/ul decrease in CD4 T-cell counts beyond the initial 12 months of ART. Other independent factors found to be significantly associated with better CD4 T-cell recovery >12 months post-ART included younger age, female sex, Indian and Malay compared to Chinese ethnicity, higher hsCRP (log), longer duration of ART, higher baseline viral load (log), higher viral load (log and time-dependent), higher baseline CD4 T-cell count and higher CD8 T-cell counts (time dependent) (Table 4).

**Table 4:** Risk factors associated with CD4 T-cell recovery >12 months period following ART initiation.

Variable	Univariate β-coefficient	*p*-value	Multivariate β-coefficient	*p*-value
Age	-3.15 (-4.05, -2.25)	<0.001*	-27.88 (-34.13, -21.60)	<0.001*
Gender				
Male				
Female	63.42 (37.22, 89.63)	<0.001*	52.87 (28.56, 77.18)	<0.001*
Ethnicity		0.004*		<0.005*
Chinese			Ref	
Indian	59.59 (20.40, 98.77)		65.90 (28.52, 103.29)	
Malay	25.20 (-3.18, 53.58)		26.93 (2.08, 51.79)	
History of AIDS-defining illness		<0.001*		<0.001*
No				
Yes	37.08 (17.51, 56.66)		30.22 (13.73, 46.72)	
Hep C antibody	1.04 (-39.10, 41.17)	0.960		
Hep B antigen	-1.99 (-39.87, 35.90)	0.918		
Number of HHV co-infections		0.003*		<0.001*
1				
2	-89.05 (-160.15, -17.94)		-160.97 (-2222.46, -99.48)	
3	-101.77 (-170.30, -32.25)		-152.27 (-211.29, -93.26)	
4	-73.70 (-142.67, -4.74)		-137.75 (-197.58, -77.92)	
hsCRP (Log)	-31.90 (24.14, 39.66)	<0.001*	18.29 (11.49, 25.09)	<0.001*
Duration of ART	1.96 (-0.76, 4.69)	0.157	10.47 (7.92, 13.02)	<0.001*
Baseline Viral Load (Log)	-23.07 (-30.01, -16.12)	<0.001*	8.38 (2.12, 14.63)	<0.001*
Viral Load (Log)	-29.04 (-46.50, -11.58)	0.001*	-35.76 (-49.33, -22.18)	<0.001*
Baseline CD4 T-cell count	0.88 (0.81, 0.96)	<0.001*	0.99 (0.92, 1.07)	<0.001*
CD8 T-cell count (Time-dependent)	0.20 (0.18, 0.22)	<0.001*	0.16 (0.14, 0.18)	<0.001*
K/T (Log) ratio	-40.27 (65.88, -14.66)	0.002*	-25.39 (-48.85, -1.94)	0.034*
**INTERACTION**				
K/T (Log) ratio X Age			-6.81 (-8.47–-5.16)	<0.001*

*Significance at p<0.05.

Abbreviation: hsCRP, C-reactive protein; ART, antiretroviral therapy; K/T ratio, ratio of kynurenine to tryptophan.

## Discussion

This is the first study to explore the influence of HHV co-infections on K/T ratio in treated HIV-infected participants and assess its impact on CD4 T-cell recovery in a developing country setting where HHV infections are highly prevalent. We found that the seroprevalences of CMV, VZV, HSV-1 and HSV-2 in HIV-infected participants were high and co-infections with multiple HHV were prevalent (97%) as previously described in other HIV studies [9,42]. Of note, all our participants reported seropositivity to at least one HHV. K/T ratio correlated with CMV and VZV IgG levels. In study participants with multiple HHV infections, co-infection involving CMV had the strongest modulating effect on K/T ratio. Increasing number of HHV co-infections in a participant and K/T ratio significantly correlated with multiple markers of immune activation and systemic inflammation. An increasing number of HHV co-infections was also independently associated with poorer CD4 T-cell recovery following 12 months of ART; however, the effect of K/T ratio on CD4 T-cell recovery was only marginal. This observation has significant implications in the developing country setting where chronic HHV co-infection is highly prevalent.

The high prevalence of concurrent HHV infection in our study is important in the context of considering the role HHV co-infection in the development of age-related complications. More specifically, the prevalence of neurocognitive and cardiovascular disorders which have both been shown to be associated with HHV serology levels [16,18], have recently been shown to be high among young treated HIV-infected participants in our setting [43].

In this study, we found CMV, VZV and HSV-2 IgG levels to positively correlate with K/T ratio which remained significant even after excluding participants with chronic hepatitis B and C co-infections (data not shown) implying that HHV was an important and persistent modulator of the IDO pathway in participants receiving long-term suppressive antiretroviral therapy. In HIV-uninfected individuals, HSV-2 and CMV have also been shown to up-regulate tryptophan metabolism, where increased IDO activity is thought to contribute to the effector antiviral mechanisms against these viruses [44]. Prior studies in HIV-infected participants have found derangements in K/T ratio to be associated with the loss of Th17 cells which promotes microbial translocation and persistent inflammation [26,45]. We also observed a strong relationship between K/T ratio and sCD14, a surrogate marker for microbial translocation and other markers of systemic inflammation (IL-6, neopterin and hsCRP), consistent with these prior studies [24]. Our data suggests that derangements in the kynurenine-tryptophan pathway may be a common marker reflecting immune dysfunction driven by both viral and bacterial antigen exposure, both of which have been shown to be associated with chronic immune activation and multiple co-morbidities in HIV [12,46].

We observed that hsCRP, IL-6 and IFN-γ were significantly higher in participants with increasing seropositivity to multiple HHV pathogens after adjustment with age, gender and ethnicity. K/T ratio however, did not differ across increasing number of HHV co-infections implying that the modulating effect of HHV on tryptophan metabolism was not additive in nature and perhaps related to the type of HHV co-infection rather than number. We then explored the effect of differing combinations of HHV seropositivity on K/T ratio given that all the HIV-infected participants in our cohort were seropositive to at least one of the HHVs tested. The combination of CMV, VZV and HSV-2 was associated with the highest derangement of the kynurenine-tryptophan pathway as expressed by the highest K/T ratio while the lowest modulating effects of this pathway were observed in participants seronegative for CMV. CMV has previously been described to be among the most immunogenic chronic viral infections [47] as it has a complex biology that evades host immune recognition [48]. In the setting of HIV, T-cell repertoires have been shown to reduce in favor of CMV-specific CD8 T-cell expansion [33] which outnumber the proportion of T-cells against other HHVs [49]. CMV has also been shown to elicit a bystander activation effect on non CMV-specific T-cells through the release of cytokine and cytokine receptor homologs [50]. These unique effects of CMV on the host immune system may explain its strong modulating effect on K/T ratio compared to the other HHVs.

Immune recovery is highly variable despite years of suppressive ART [2] and associated with serious non-AIDS events [3]. In this study, we explored the potential role of HHV seroprevalence and persistent kynurenine-tryptophan dysregulation on CD4 T-cell recovery. HHV seroprevalence quantified as an increasing number of HHV co-infections in a participant was significantly associated with in lower CD4 T-cells following 12 months of suppressive therapy, confirming findings from a prior cross-sectional study which showed reduced immune reconstitution in treated HIV-infected participants co-infected with CMV [32]. Despite a statistically significant association in multivariate analysis, the impact of K/T ratio on changes in CD4 T-cells post-ART was relatively small and clinically insignificant. A prior study in Uganda assessing the impact of K/T ratio measured at 12 months post-ART on subsequent CD4 T-cell recovery also reported a significant but small decrease in CD4 T-cell counts (2 cells/ul/month) with each doubling of K/T ratio [28]. Downstream catabolites of tryptophan metabolism such as kynurenine and picolinic acid have been shown to inhibit T-cell proliferation [51]. Kynurenine has recently been shown to inhibit IL-2 mediated signaling in memory CD4 T-cells via increased reactive oxygen species production, making them susceptible to Fas-mediated apoptosis [31]. Additionally, HHVs such as CMV have also been associated with bystander activation-induced cell death [52] which could further contribute to HHV-associated CD4 T-cell loss independent of its effect on tryptophan metabolism. Indeed, we found higher systemic inflammation levels with increasing number of HHV co-infections in this cohort. Periodic subclinical reactivation of HHV during treated HIV disease may also provide increased target cells for HIV infection [53] and promote further CD4 T-cell loss. Thus, strategies to control HHV infection/re-activation rather than IDO inhibition in HIV may be beneficial in optimizing immunological responses to ART. Although a prior study with valgancyclovir [54] in ART treated participants did not show a significant benefit in improving CD4 T-cell counts despite an impact on CD8 T-cell activation, no studies have so far been conducted in a resource limited setting where background levels of immune activation and IDO activity are higher despite ART [28,55] and the interaction between HHV and HIV on clinical outcomes are likely to be more significant. Our recovery model assessed from 0–12 months post-ART showed that an increase in CD4 T-cell counts were associated with K/T ratio increase. We speculate that the associations assessed during this period may largely reflect the redistribution of CD4 T-cells from the lymph nodes rather than *de novo* CD4 T-cell production and thus may not reflect the true biological relationship between these parameters [38].

There were several important limitations to this study. First, this is a cross-sectional study and hence, we cannot infer any causal relationship from the associations found in this study. Secondly, we used IgG antibody levels as a surrogate of HHV infections and did not measure HHV DNA levels as done in some studies [10,11]. We also cannot exclude that the increased HHV antibody levels found could have partially been contributed by an increase in global B cell activation as previously described in HIV [56] and assessments of cellular proliferative index of total non-specific B cells and total IgG plasma levels would have helped discern this [57]. Nevertheless, numerous studies assessing the modulating effect of HHV on host immune responses [33] and clinical outcomes [8,16] have previously utilized IgG as a surrogate for HHV infection and found these to reliably correlate with morbidity and mortality outcomes in HIV-infected and uninfected participants. Additionally, we only measured HHV IgG levels at a single time-point post-ART and thus acknowledge that the potential influence of the duration of HHV infection on K/T ratio and immune reconstitution could not be reliably assessed in this study. Thirdly, we only focused on CMV, VZV, HSV-1 and HSV-2 but not the other HHVs in our study, as these four are the common HHV infections in HIV-infected participants [42]. Fourthly, recent studies have described the influence of genetic factors associated with K/T ratio in treated HIV [58]. The influence of these host factors were not assessed in our study and thus we cannot be certain of their potential modifying effect on the associations between HHV seropositivity and K/T ratio assessed in our study. Furthermore, data from our study was from a single tertiary site with an over-representation of ethnic Chinese participants and thus our findings may not be generalizable to the HIV population in Malaysia. Finally, the number of participants who were seronegative for specific HHV infections particularly CMV was quite small and this could have impacted our ability to demonstrate significant differences in measured markers of immune activation between seropositive and negative participants.

## Conclusion

In conclusion, co-infection with multiple HHV is common in treated HIV-infected participants in the developing country setting and significantly impacts CD4 T-cell recovery. Although HHV infections and specifically CMV was a strong modulator of IDO activity, the influence of this pathway on reduced CD4 T-cell recovery in HHV-HIV co-infected participants were clinically insignificant.

## Supporting information

S1 Table Comparison of immune activation markers between each human herpes virus (HHV) seronegative and seropositive. (DOC)
